# Sodium-Glucose Cotransporter 2 Inhibitors During Cancer Therapy: Benefits, Risks, and Ongoing Clinical Trials

**DOI:** 10.1007/s11912-024-01577-8

**Published:** 2024-07-11

**Authors:** Nichanan Osataphan, Husam Abdel-Qadir, Agnieszka Maria Zebrowska, Anna Borowiec

**Affiliations:** 1 Division of Cardiology, Ted Rogers Program in Cardiotoxicity Prevention, Peter Munk Cardiac Center, Toronto General Hospital, University Health Network, University of Toronto, Toronto, ON Canada; 2 Division of Cardiology, Department of Internal Medicine, Faculty of Medicine, Chiang Mai University, Chiang Mai, Thailand; 3 Women’s College Hospital, Toronto, ON Canada; 4 Department of Cancer & Cardio-Oncology Diagnostics, The Maria Sklodowska-Curie National Research Institute of Oncology, Roentgena Str 5, 02-781 Warsaw, Poland; 5 Unit for Screening Studies in Inherited Cardiovascular Diseases, The Cardinal Stefan Wyszynski National Institute of Cardiology, Warsaw, Poland

**Keywords:** CTRCD, Cardiotoxicity, Cardioprotection, SGLT2i, Cancer, Cardiooncology

## Abstract

**Purpose of review:**

The goal of this paper is to summarize the data pertaining to the use of sodium-glucose cotransporter-2 inhibitors (SGLT-2i) for the prevention of cardiotoxicity in patients receiving anthracyclines for cancer treatment. We discuss the potential efficacy of this class of medications, incorporating insights from existing literature and ongoing studies.

**Recent findings:**

SGLT2i are a class of medications which were initially developed for treatment of Type 2 diabetes and later extended to treat heart failure with reduced and preserved ejection fraction regardless of diabetes status. There remains a need for effective and safe treatments to preventing cardiotoxicity in anthracycline-treated patients. It has been proposed that SGLT2i may provide protection against the cardiotoxic effects of anthracyclines. Some of the proposed mechanisms include beneficial metabolic, neurohormonal, and hemodynamic effects, renal protection, as well as a decrease in inflammation, oxidative stress, apoptosis, mitochondrial dysfunction and ion homeostasis.

**Summary:**

There is emerging evidence from basic science and observational studies that SGLT2i may play a role in the prevention of chemotherapy-induced cardiotoxicity. Randomized controlled trials are needed to conclusively determine the role of SGLT2 inhibitors as a cardioprotective therapy in patients receiving anthracyclines for the treatment of cancer.

## Introduction

Sodium-glucose cotransporter-2 inhibitors (SGLT2i) were initially developed for the treatment of type 2 diabetes. It was subsequently demonstrated that SGLT2I are efficacious in reducing cardiovascular events in heart failure (HF) patients with reduced and preserved left ventricle ejection fraction (LVEF) with and without diabetes [1–3]. Accordingly, the 2023 ESC updated guidelines for the treatment of HF, SGLT2i are recommended for all patients with HF, regardless of left ventricular ejection fraction [3, 4].

Cancer therapy-related cardiovascular toxicity (CTR-CVT) is defined as either one of: chemotherapy-related cardiac dysfunction (CTRCD), myocarditis, vascular toxicity, hypertension, arrhythmias, or QT prolongation [5]. Various cancer therapies carry the risk of cardiotoxicity, including anthracyclines, immune checkpoint inhibitors, kinase inhibitors, and radiotherapy. There remains a need for treatments to reduce the risk of CTR-CVT. In vitro and observational studies suggest that the use of SGLT2i during anthracycline therapy may be a promising cardioprotective strategy.

### Anthracycline Related Cardiac Dysfunction

Anthracycline cardiotoxicity is characterized by decreased LVEF, which may lead to symptomatic heart failure. The cardiotoxicity of anthracyclines usually manifests in the year after treatment [6], although clinical heart failure may be first recognized years after treatment completion [7]. Anthracyclines induce cardiotoxicity through a dose dependent, cumulative and progressive process.

Ideally, interventions aimed at preventing anthracycline cardiotoxicity should primarily target the underlying mechanisms. However, the mechanisms of anthracycline-induced cardiotoxicity are complex and remain controversial. A leading hypothesized mechanism involves the binding of anthracyclines to topoisomerase IIβ within the nucleus of cardiomyocytes, resulting in DNA double-strand breaks which lead to cardiomyocyte apoptosis [8]. Oxidative stress and mitochondrial dysfunction are also hypothesized to play an important role in anthracycline-induced cardiotoxicity [9, 10]. Anthracyclines increase reactive oxygen species (ROS) production through redox-cycling leading to the generation of semiquinone compound [11]. Excessive production of ROS can result in the oxidation of proteins and signaling molecules, as well as damage to mitochondrial DNA [10], leading to the opening of the mitochondrial permeability transition pore (mPTP) and activation of the apoptosis pathway [12].

Anthracyclines may also directly suppress the activity of electron transport chain complexes within the mitochondria leading to a reduction in ATP production [13]. Since the heart has high mitochondrial density, the disruption of mitochondrial bioenergetics by anthracyclines can substantially impair cardiac function [11]. Anthracyclines may also interfere with autophagy, which is a conserved process aimed at maintaining cell and tissue homeostasis by degradation of damaged organelles or non-functioning proteins [14]. Anthracyclines have been demonstrated to enhance autophagic initiation and autophagosome formation while inhibiting autophagic flux [15]. Inhibiting lysosomal acidification prevents the completion of autophagic flux, resulting in the accumulation of autolysosomes and the generation of ROS, ultimately leading to cardiotoxicity [16]. The Signal transducer and activator of transcription 3 (STAT3) pathway, which is important for cardiomyocyte survival, is also reported to be suppressed by doxorubicin [17].

### Current Approaches for Primary Prevention of Anthracycline Cardiotoxicity

Some of the currently used strategies to prevent CTRCD are summarized in Table 1. Primary prevention interventions are more likely to have a favorable risk–benefit balance in people who are at elevated risk of cardiotoxicity. The Heart Failure Association-International Cardio-Oncology Society (HFA-ICOS) risk tool is currently recommended for pre-treatment risk assessment [7]. This tool integrates clinical risk factors, baseline LVEF, and baseline biomarkers to determine an individual’s risk. It also allows clinicians to manage modifiable risk factors and develop appropriate monitoring strategies. Alternative chemotherapeutic agents can also be considered for people at high risk of cardiotoxicity if that can be pursued with minimal loss of anti-cancer efficacy.

**Table 1:** Preventive strategies for CTRCD and their implementations

Strategies	Treatment/intervention	Implementation
Identification of individual risk	- Risk stratification with HFA-ICOS	- Optimize cardiovascular risk factors before, during and after cardiotoxic treatment - Consider more frequent follow up in patients with high or very high-risk for CTRCD
Anthracycline delivery methods	- Dexrazoxane - Liposomal doxorubicin	- Consider dexrazoxane in patients with advanced/ metastatic breast cancer already received a minimum cumulative anthracycline dose of 300—mg/m^2^ of doxorubicin or equivalent - Consider dexraxozane and liposomal doxorubicin in patients with high or very high risk for CTRCD receiving anthracyclines
Pharmacological intervention	**Neurohormonal antagonists** - Beta-blocker - ACEI/ARB **Other agents** - Statins - SGLT2i	- Consider ACEI/ARB and beta-blocker in patients with high or very high risk for CTRCD - Consider statin in patients with high or very high risk for CTRCD regardless of LDL level - May consider SGLT2i in patients with DM undergoing cardiotoxic treatment
Non-pharmacological intervention	- Maintaining adequate physical activity - Smoking cessation - Restricting alcohol consumption to 100 g per week	- Regular exercise to maintain cardiorespiratory fitness and quality of life

*ACEI* Angiotensin converting enzyme inhibitor, *ARB* Angiotensin receptor blocker, *CTRCD* Chemotherapy-related cardiac dysfunction, *HFA-ICOS* Heart Failure Association-International Cardio-Oncology Society, *SGLT2i* Sodium-glucose cotransporter-2 inhibitor

Most pharmacological interventions that have been considered for the primary prevention of CTRCD have been adapted from the management of heart failure. Neurohormonal blockade medications, including beta-blockers, angiotensin-converting enzyme inhibitors, angiotensin receptor blockers, and mineralocorticoid receptor antagonists, have been explored as a primary prevention strategy for patients receiving anthracyclines. While these agents have shown substantial benefit in the setting of heart failure with reduced ejection fraction (HFrEF), they have demonstrated minimal benefit in patients receiving cardiotoxic chemotherapy [18–20]. The modest or negative outcomes observed in this population may be attributed to the lower degrees of neurohormonal activation compared to patients with HFrEF. Importantly, neurohormonal blockade is not part of the recognized pathways for the development of anthracycline cardiotoxicity.

Statins are drugs with pleiotropic properties that do not influence hemodynamic changes, making them attractive options for preventing cardiotoxicity for people actively undergoing chemotherapy. After observational studies suggested benefits [21], several RCTs tested the efficacy of statins in preventing CTRCD in anthracycline-treated patients [22–24]. In the PREVENT and SPARE-HF studies, administering atorvastatin 40 mg during anthracycline treatment did not mitigate the reduction in LVEF following anthracycline treatment [22, 24]. Conversely, the STOP-CA trial, which involved patients with lymphoma, showed that atorvastatin significantly reduced the incidence of cardiac dysfunction when compared with the placebo [23].

### Current Indications for the Use of SGLT2 Inhibitors

Before considering novel indications for the use of SGLT2I, it is important that people undergoing cardiotoxic therapy are treated with SGLT2i if they meet current indications. The 2023 ESC guidelines for the management of cardiovascular disease in patients with diabetes recommend that individuals with DM and atherosclerotic cardiovascular disease (ASCVD) or those at high risk for ASCVD should be prescribed SGLT2i with proven cardiovascular benefits, including dapagliflozin, empagliflozin, canagliflozin, and sotagliflozin [25].

Given their impressive impact on reducing heart failure hospitalizations in people with diabetes, empagliflozin and dapagliflozin were further studied in patients with symptomatic heart failure with reduced and preserved ejection fraction [2–4, 26]. These studies involved patients both with and without diabetes and demonstrated positive results, primarily characterized by a reduction in the risk of heart failure hospitalization. Empagliflozin and dapagliflozin are now recommended as class I indication for symptomatic HFrEF, HFmrEF and HFpEF [27]. Furthermore, SGLT2i have demonstrated a beneficial impact on renal outcomes, possibly attributed to their ability to lower intraglomerular pressure and reduce albuminuria [28, 29]. Accordingly, SGLT2i are recommended for patients with chronic kidney disease (CKD) and diabetes to mitigate the risk of declining kidney function [30]. However, it should be emphasized that the results of the EMPA-KIDNEY trial indicate a similar beneficial effect also in patients without diabetes [31].

### The Potential Mechanisms of Cardioprotection by SGLT2i

SGLT2 has no expression in the human heart [32]. Thus, any potential cardioprotective benefit from SGLT2i is likely due to indirect effects of SGLT2i, possibly systemic hemodynamic and/ or metabolic effects. The proposed mechanisms responsible for the cardiorenal protection of SGLT2i are multifaceted, encompassing both hemodynamic and metabolic effects [28]. SGLT2i promote natriuresis by inhibiting sodium reabsorption in the proximal convoluted tubule. This, in turn, intensifies tubuloglomerular feedback, subsequently lowering intraglomerular pressure and thereby preserving kidney function [33]. The reduction in extracellular volume due to natriuresis also leads to a decrease in systemic blood pressure and preload, which is the proposed hemodynamic mechanism for reducing heart failure with SGLT2i [33].

Treatment with SGLT2i is associated with an improvement in myocardial energy metabolism [34] by increasing elevated levels of circulating ketone bodies, specifically beta-hydroxybutyrate [35]. SGLT2i induce a metabolic state that resembles some aspects of the accelerated starvation response by increasing gluconeogenesis and free fatty acid oxidation [34]. Ketone bodies are often labeled as "super-fuels" due to their superior oxygen efficiency compared to fatty acids [36]. By increasing ketone body production, SGLT2i may enhance cardiac energy metabolism and improved cellular function at mitochondrial level [37]. Furthermore, the oxidation of ketone bodies is independent of the oxidation rates of fatty acids or glucose [38]. Therefore, ketone bodies offer an additional source of fuel for cardiac metabolism. Increased ketone metabolism is observed in patients with heart failure, so a transition toward ketone body metabolism is considered an adaptive response in the failing heart [38, 39]. Accordingly, this metabolic shift associated with SGLT2i is a leading proposed cardioprotective mechanism of this drug.

In animal and in vitro studies of doxorubicin models, have raised alternative hypotheses as to how SGLT2i may prevent anthracycline cardiotoxicity. There is some evidence indicating that SGLT2i may directly bind to glucose transporters (GLUT) within cardiomyocytes, thereby reducing glycolysis and improving oxidative phosphorylation in mitochondria [40]. Additionally, they may inhibit the Na/H + exchanger within cardiomyocyte to alleviate sodium and calcium overload, leading to improved cardiac function in mouse models of heart failure [41]. Another potential cardioprotective mechanism of SGLT2 inhibitors is a reduction of ROS production leading to decreased cardiomyocyte apoptosis [17, 42, 43]. Phosphatidylinositol 3-kinase (PI3K)/protein kinase B (AKT) is an important regulator of the survival of cardiomyocytes [44]. SGLT2i can activate PI3K/AKT signaling in cardiomyoblasts following doxorubicin treatment, resulting in a reduction in ROS production and the preservation of mitochondrial function [42]. STAT3 has been demonstrated to participate in various cardioprotective mechanisms, and the restoration of STAT3 by dapagliflozin was associated with a reduction in cardiomyocyte apoptosis [17, 45]. There is some evidence to suggest that SGLT2i may mitigate cardiotoxicity by regulating autophagy [46]. In the mouse model treated with doxorubicin, it was observed that empagliflozin increased autophagic flux, decreased the accumulation of autolysosomes, and protected the heart from cardiotoxicity [46].

By reducing ROS production, apoptosis, preserving mitochondrial function, and regulating autophagy, the suspected cardioprotective effects of SGLT2i may target the specific mechanisms of anthracycline-induced cardiotoxicity [9, 10]. Overall, the administration of SGLT2i in doxorubicin models contributed to the preservation of cardiomyocyte morphology, a reduction in myocardial fibrosis, an improvement in cardiac contractility, and the reversal of cardiac remodeling [47–50]. The entirety of these cardioprotective effects of SGLT2 inhibitors culminates in the reversal of cardiac remodeling, as demonstrated by a reduction in left ventricular mass and an improvement in left ventricular ejection fraction [51, 52].

### Observational Data Supporting the Use of SGLT2I for the Prevention of Cardiotoxicity

At the time of this review, we identified 5 observational studies of SGLT2i in cardio-oncology (Table 2). The first study by Gongora et al. was a retrospective study of patients with DM and cancer who were treated with anthracyclines [53]. They studied 32 patients treated with SGLT2i, who were matched to 96 controls on age, sex, and the starting date of anthracycline treatment. They observed that the use of SGLT2i was associated with a lower risk of mortality (9% in SGLT2i vs. 43% in non-SGLT2i, *p* < 0.001) and a composite of heart failure incidence, new cardiomyopathy (defined as > 10% decline in ejection fraction to < 53%), heart failure hospitalizations, and significant arrhythmias (3% in SGLT2i vs. 20% in non-SGLT2i, *p* = 0.025). Another investigation conducted by Chiang et al. further substantiated the advantages of SGLT2i in individuals with both cancer and diabetes mellitus [54]. Employing propensity score matching, 878 patients utilizing SGLT2i were carefully matched with an equal number of patients not using SGLT2i. The results indicated that SGLT2i usage was correlated with a decrease in heart failure hospitalizations and overall mortality. It is worth noting, however, that only 8% of the patients in this study received anthracycline.

**Table 2:** Summary of observational data of SGLT2i in Cardio-oncology

Author	Population	Trial design	Outcomes
Gongora et al. [53]	Cancer and DM treated with anthracyclines	1:3 ratio matched by age, sex, and anthracyclines starting date SGLT2i (*n* = 32) Non-SGLT2i (*n* = 96)	-**Composite of cardiovascular outcomes** (HF incidence, new cardiomyopathy, HF hospitalizations, and significant arrhythmias): 3% in SGLT2i vs 20% in non-SGLT2i (*p* = 0.025) -**Overall mortality:** 9% in SGLT2i vs 43% in non-SGLT2i (*p* < 0.001)
Chiang et al. [54]	Cancer and T2DM (8% received anthracyclines)	1:1 propensity score matching for demographic data, comorbidities, use of cardiovascular medications and the type of cancer therapy SGLT2i (*n* = 878) Non-SGLT2i (*n* = 878)	-Hospitalization for HF: SGLT2i associated with HR:0.28; 95%CI: 0.11–0.77; *p* = 0.013 -Overall survival: SGLT2i associated with HR:0.35; 95%CI:0.28–0.43; *p* < 0.001
Abdel-Qadir et al. [55]	Cancer and DM treated with anthracyclines, age > 65 years, and no prior HF	Estimating propensity score for SGLT2i use SGLT2i (*n* = 99) Non-SGLT2i (*n* = 834)	-**HF hospitalization:** SGLT2i associated with HR:0; 95%CI: N/A; *p* < 0.001 (no patient in SGLT2i developed HF hospitalization) -**Incidence HF:** non-difference, HR:0.55; 95%CI: 0.23–1.31; *p* = 0.18 **-Any CVD diagnosis:** non-difference, HR:0.39; 95%CI: 0.12–1.28; *p* = 0.12 -**Mortality:** non-difference, HR:0.63; 95%CI: 0.36–1.11; *p* = 0.11
Avula et al. [56]	Cancer and T2DM exposed to cardiotoxic treatment with a subsequent diagnosis of cardiomyopathy or HF Excluded patients with a diagnosis of acute coronary syndrome, had CABG or PCI	1:1 propensity score matching for demographics, comorbidities, and medications GDMT and SGLT2i (*n* = 640) GDMT only (*n* = 640)	**-Acute HF exacerbation:** SGLT2i associated with OR:0.483; 95%CI: 0.36–0.65; *p* < 0.001 **-All-cause mortality:** SGLT2i associated with OR:0.296; 95%CI: 0.22–0.40; *p* = 0.001 **-All-cause hospitalization or ED visits:** SGLT2i associated with OR:0.479; 95%CI: 0.383–0.599; *p* < 0.001 **-Atrial fibrillation/flutter:** SGLT2i associated with OR:0.397; 95%CI: 0.213–0.737; *p* = 0.003 **-Acute kidney injury:** SGLT2i associated with OR:0.486; 95%CI: 0.382–0.619; *p* < 0.001 **-Need for renal replacement therapy:** SGLT2i associated with OR:0.398; 95%CI: 0.189–0.839; *p* = 0.012
Hwang et al. [57]	Cancer with T2DM and non-DM undergoing anthracycline therapy	Two propensity score matched cohorts T2DM with SGLT2i (*n* = 779) vs Non-DM (*n* = 7800) T2DM with SGLT2i (*n* = 779) vs T2DM without SGLT2i (*n* = 2337)	**-Composite outcome (HF hospitalization, AMI, ischemic stroke, death)** **T2DM with SGLT2i vs Non-DM: SGLT2i associated with adjusted HR:** 0.35; 95%CI:0.25–0.51; *p* < 0.05 **T2DM with SGLT2i vs T2DM without SGLT2i: SGLT2i associated with adjusted HR:** 0.47; 95%CI:0.32–0.69; *p* < 0.05

*AC* Anthracyclines, *AMI* Acute myocardial infarction, *CABG* Coronary artery bypass graph, *DM* Diabetes mellitus, *ED* Emergency department, *GDMT* Guideline-directed medical therapy, *HF* Heart failure, *HR* Hazard ratio, *OR* Odds ratio, *PCI* Percutaneous coronary intervention, *SGLT2i* Sodium glucose transporter 2 inhibitor, *T2DM* Type 2 diabetes mellitus

A subsequent, larger study focused on a higher-risk group of individuals aged > 65 years with cancer and DM treated with anthracyclines [55], of whom 99 were users of SGLT2i and 834 were treated for diabetes without SGLT2is. Propensity score methods were used to weigh individuals by the average treatment effects for the treated to reduce baseline differences. It was observed that SGLT2i significantly reduced the risk of HF hospitalization (HR 0, as no patient in the SGLT2i group had HF hospitalization) with a non-significant trend towards a decreased risk of new HF diagnoses (in- or out-of-hospital), documentation of any CVD in future hospitalizations, and overall mortality.

In addition, Avula et al. suggested that SGLT2i may also improve outcomes in cancer patients exposed to cardiotoxic treatment who subsequently developed CTRCD and HF [56]. Patients aged ≥ 18 years with a history of type 2 DM, cancer, and exposure to potentially cardiotoxic antineoplastic therapies, with a subsequent diagnosis of cardiomyopathy were identified. Propensity scores were used to match 640 patients in the guideline-directed medical therapy (GDMT) plus SGLT2i group and 640 patients in GDMT without SGLT2I. In this study, taking SGLT2i together with GDMT reduced acute HF exacerbation, mortality, all-cause hospitalization or emergency department visits, atrial fibrillation/flutter relative to patients receiving GDMT without SGLT2I.

In the latest study utilizing South Korean Nationwide data [57], 779 patients treated with anthracyclines for newly diagnosed cancer were matched using propensity scores to 2337 individuals with DM not using SGLT2i, and to individuals without DM (*n* = 7800). Patients with DM using SGLT2i were observed to have a lower risk of a composite of adverse cardiovascular outcomes (heart failure hospitalization, ischemic stroke, acute myocardial infarction, and death) compared to both comparator groups.

In summary, all the observational data available to date suggest the potential cardiovascular benefits of SGLT2 inhibitors in cancer patients undergoing anthracycline chemotherapy. However, there are some limitations when interpreting the results from the observational studies, given the unmeasured residual confounders or selection bias. All patients in these studies have DM, and whether the cardioprotective effects against cardiotoxic therapies will be the same in non-DM individuals requires further investigation. These results endorse the need for randomized trials to investigate the impact of SGLT2i on cardiac outcomes in patients receiving anthracycline treatment.

Interestingly, emerging evidence suggests that SGLT2 inhibitors may have anticancer effects and can potentially slow tumor growth in mouse models of breast and colon cancer [12, 58]. These effects include the reduction of glucose uptake into cancer cells and the regulation of various gene and protein expressions. However, a meta-analysis concluded that SGLT2i exposure neither decreased nor increased the overall risk of cancer and cancer-related mortality [59]. Importantly, there are no emergent safety concerns associated with the use of SGLT2i in these observational studies. SGLT2i did not increase the risk of urinary tract infection, lower limb amputation or genital infection [53, 56]. However, adverse effects may be underestimated since the observational studies focused on prevalent users (thus excluding people who stopped drugs because of adverse effects). Collectively, these observational data cannot be used to promote off-label use of SGLT2i for cardiotoxicity prevention and raises the need for RCTs involving cancer patients.

## Randomized clinical trials investigating the role of SGLT2 Inhibitors in Preventing Cardiotoxicity During Cancer Therapy

**Fig. 1:**
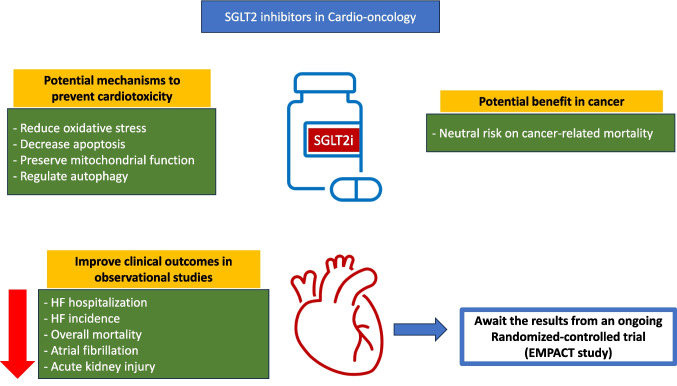
The potential cardiovascular benefits of SGLT2 inhibitors in cancer patients. SGLT2i; sodium glucose transporter 2 inhibitor, HF; heart failure, EMPACT; Empagliflozin in prevention of chemotherapy-related cardiotoxicity

Due to the absence of human clinical studies evaluating the efficacy of SGLT2i in preventing cardiovascular events, a randomized clinical trial was initiated at the The Maria Sklodowska-Curie National Research Institute of Oncology in Warsaw, Poland. Empagliflozin in prevention of chemotherapy-related cardiotoxicity (EMPACT, NCT05271162) is a double-blinded, randomized, placebo-controlled study aimed at assessing the effectiveness, and safety of 10 mg empagliflozin administered during anthracycline treatment for the prophylaxis of cardiotoxicity/CTRCD in patients without heart failure and with preserved left ventricle ejection fraction greater than 50%. EMPACT is a multicenter, prospective study which is currently actively recruiting with a goal of enrolling a population of approximately 220 patients with cancer qualified for treatment with high doses of anthracyclines (doxorubicin ≥ 240 mg/m^2^ or epirubicin ≥ 360 mg/m^2^). Participants will receive either 10 mg empagliflozin or placebo every day for a year and will be followed for an additional 12 months. The primary objective of the EMPACT study is to assess whether prophylactic SGLT2i may prevent a reduction in LVEF after high doses anthracyclines, as evaluated by serial echocardiography on each visit and cardiovascular magnetic resonance (CMR) performed at randomization and on its completion. The secondary composite endpoint includes all-cause death, cardiovascular (CV) death, myocardial infarction and ischemic stroke. Additional secondary outcome measures include structural myocardial alterations assessed by CMR, decrease in GLS (global longitudinal strain) in echocardiography and changes in cardiac biomarkers. The EMPACT study received financial support from the Polish Medical Research Agency (Fig. 1).

## Conclusion

There remains a strong need for interventions to reduce the risk of CTRCVT in cancer. SGLT2i constitute a novel and promising treatment option for this purpose. There are many potential mechanisms that can lead to the development of heart failure in cancer patients, and the mechanism of action of SGLT2i may attenuate some of these pathways. There remains much to be understood regarding the impact of SGLT2i on the prevention of cardiotoxicity. An ongoing randomized controlled trial in this area are highly anticipated among oncologists, cardiologists, and cardio-oncologists.

## Data Availability

No datasets were generated or analysed during the current study.
